# A comparative analysis of autograft choices of anterior cruciate ligament reconstruction and their effects on muscle strength and joint biomechanics

**DOI:** 10.3389/fspor.2024.1444465

**Published:** 2025-01-27

**Authors:** Wiem Issaoui, Ismail Dergaa, Hatem Ghouili, Abdelfatteh El Omri, Noomen Guelmami, Philippe Chomier, Mourad Ghrairi, Helmi Ben Saad, Wassim Moalla

**Affiliations:** ^1^ High Institute of Sport and Physical Education, University of Sfax, Sfax, Tunisia; ^2^ Health Medical Services (HMS) FIFA Medical Centre of Excellence Dubai, Dubai, United Arab, Emirates; ^3^ Research Unit “Sport Sciences, Health and Movement”, Higher Institute of Sports and Physical Education of Kef, University of Jendouba, El Kef, Tunisia; ^4^ Department of Preventative Health, Primary Health Care Corporation (PHCC), Doha, Qatar; ^5^ Departement of Biological Sciences, High Institute of Sport and Physical Education Ksar Saïd, University of Manouba, Manouba, Tunisia; ^6^ Clinical Advancement Department, Hamad Medical Corporation, Doha, Qatar; ^7^ Service of Physiology and Functional Explorations, Farhat Hached Hospital, University of Sousse, Sousse, Tunisia; ^8^ Research Laboratory LR12SP09 “Heart Failure”, Farhat Hached Hospital, University of Sousse, Sousse, Tunisia; ^9^ Laboratory of Physiology, Faculty of Medicine of Sousse, University of Sousse, Sousse, Tunisia; ^10^ Research Laboratory Education, Motricité, Sport et Santé (EM2S) LR19JS01, High Institute of Sport and Physical Education of Sfax, University of Sfax, Sfax, Tunisia

**Keywords:** ACL, exercise therapy, functional performance, knee, orthopedic surgery, postoperative care, rehabilitation, sports medicine

## Abstract

**Introduction:**

Anterior cruciate ligament reconstruction (ACLR) is crucial to restore knee stability and function after ACL injuries, especially in physically active individuals. Despite advances in surgical techniques and rehabilitation protocols, the choice of autograft has a significant impact on postoperative recovery, particularly on muscle strength and joint biomechanics. In this study, the effects of four autografts are investigated: Iliotibial band (ITB), combined ITB and hamstring tendon (ITB + HT), hamstring tendon (HT) and bone-tendon-bone (BTB) on quadriceps and hamstring peak torque (QPT and HPT) recovery and hamstring to quadriceps ratio (H:Q) to assess knee stability and function.

**Methods:**

Forty-two active males (mean ± standard deviation of age: 31.5 ± 6.1 years, height: 177 ± 6 cm, weight: 76 ± 11 kg, body mass index: 24.5 ± 2.2 kg/m²) with primary ACL ruptures were allocated to the four graft groups (ITB: *n* = 16, ITB + HT: *n* = 12, HT: *n* = 7, BTB: *n* = 7) and underwent a standardized rehabilitation protocol. Quadriceps and hamstring peak torque (QPT and HPT, respectively) as indicators of isokinetic muscle strength were assessed both postoperatively and follow-up after approximately six months (mean 6.29 ± 1.70 months)

**Results:**

Significant differences in QPT and HPT recovery between the healthy and injured legs were found in all graft groups (*P* < 0.001). The BTB group showed the largest QPT deficit between healthy and injured legs (Δ = 133.4 Nm, Cohen's *d* = 8.05) and HPT deficit (Δ = 41.1 Nm, Cohen's *d* = 4.01). In contrast, the ITB + HT group showed the smallest deficits in QPT (Δ = 22.5 Nm, Cohen's *d* = 0.73) and HPT (Δ = 13.5 Nm, Cohen's *d* = 1.21). The BTB group also showed the largest deviation in H:Q ratios (Δ = −0.23, Cohen's *d* = 2.70), while the HT group showed a more balanced recovery with smaller significant deficits in H:Q ratios (Δ = −0.07, Cohen's *d* = 0.46).

**Conclusion:**

The BTB graft showed the most pronounced variations in QPT and HPT between healthy and injured legs in the short term, indicating the importance of longitudinally monitoring knee stability to determine the best autograft choice for ACLR. While all graft types contribute to muscle strength recovery, the HT graft may provide advantages in balancing muscle strength and potentially enhancing knee stability.

## 1 Introduction

The anterior cruciate ligament (ACL) is a key structure in the knee joint that provides stability and allows a wide range of movements, especially during high-intensity sports activities (1, 2). The ACL stabilizes the knee by counteracting anterior tibial translation and controlling internal rotation (3, 4). Structurally, it consists of the anteromedial and posterolateral bundles, which work together to maintain knee stability throughout the range of motion (5, 6). Given their importance to knee function, ACL injuries are common and particularly problematic in athletes. They often lead to pain, instability and functional limitations and represent a major challenge for orthopedics and sports medicine (7, 8).

ACL injuries are often caused by non-contact mechanisms, such as rapid deceleration, dynamic knee valgus and rotational forces during movements such as landings or rapid changes in direction (9, 10). Proper diagnosis of ACL injuries is essential to assess the extent of the injury and develop an effective treatment plan (11–13). Treatment options include conservative management with structured physiotherapy or surgical intervention through ACL reconstruction (ACLR) (14, 15). For individuals with high physical demands, ACLR is generally the preferred approach as it effectively restores stability and function to the knee (16, 17). Over the years, ACLR techniques have evolved with a focus on refining graft selection, tunnel placement, graft tensioning, and fixation methods to optimize surgical outcomes (18, 19). Although both autografts and allografts are used, autografts are preferred due to their superior healing properties (20, 21).

Each type of autograft in ACLR has unique clinical implications regarding recovery and potential complications. The bone-tendon-bone (BTB) autograft is often favored for its strong fixation properties and high stability, making it suitable for athletes who want to return to sports quickly. However, it can lead to anterior knee pain and morbidity at the donor site due to the involvement of the patellar tendon (22). Hamstring tendon graft (HT) generally results in fewer donor site complications and less postoperative pain, but can lead to hamstring tendon weakness and a potentially higher graft elongation rate, which affects stability (23). The iliotibial band (ITB) graft and combined ITB + HT grafts offer additional options: ITB grafts have high resilience and may benefit younger patients (24), while the combination of ITB + HT may balance the strengths of both grafts, although recovery time may be prolonged due to the larger donor area (25). Understanding each autograft option's specific benefits and complications allows for more informed decisions regarding ACLR, optimizing muscle recovery and functional outcomes while minimizing adverse effects.

After ACLR, the muscles surrounding the knee, particularly the quadriceps and hamstrings, often have strength deficits due to the surgical trauma, graft harvest and postoperative inactivity. The quadriceps in particular can show considerable weakness, which can persist even after rehabilitation. Hamstring strength can also be affected, especially if a HT graft is used, although the effects vary depending on the type of graft chosen, e.g., the BTB (26). Rehabilitation is crucial for recovery, with isokinetic training at a constant movement speed proving effective in enhancing muscle strength and functional outcomes (27). The integration of isokinetic training into rehabilitation has been associated with improved quadriceps and hamstring strength, allowing a safe and effective return to sport (28, 29). Assessment of recovery outcomes through objective measures, such as isokinetic strength measurements, and patient-reported outcomes (e.g., International Knee Documentation Committee and Lysholm scores) is crucial for establishing rehabilitation protocols and monitoring treatment success (14, 30).

Despite advances in ACLR and rehabilitation protocols, several research gaps remain. Long-term studies are needed to better understand how different graft types affect muscle recovery, as most existing studies focus on short-term outcomes (15, 31). In addition, there are still questions about the most effective rehabilitation approaches, including the integration of isokinetic training and neuromuscular control programs, to achieve optimal recovery and minimize the risk of re-injury (32, 33). Indeed, both graft selection and postoperative rehabilitation are crucial in influencing muscle strength recovery following ACL reconstruction (32, 33).

Therefore, this study aimed to address critical gaps in ACLR management. Unlike previous research that focused primarily on short-term outcomes, this study followed patients for up to six months to (1) compare the effectiveness of four commonly used autograft types (ITB, ITB + HT, HT, and BTB) on quadriceps and hamstring peak torque recovery (QPT and HPT) after surgery, and (2) analyze the hamstring-to-quadriceps (H:Q) ratio in the different autograft groups to evaluate their potential impact on knee stability and function. We hypothesized that ACLR would improve isokinetic muscle strength six months post-surgery and proposed that the HT graft would be the most effective choice for balanced knee muscle recovery.

## 2 Methods

### 2.1 Study design

This study employed a prospective cohort design to thoroughly evaluate the role of isokinetic muscle strength assessment in patients undergoing ACLR with different grafts. This study was performed in FIFA Medical Centre of Excellence (Dubai, United Arab Emirates) from January 2020 to December 2023. This Study followed the ethical statements of the Declaration of Helsinki. Approval from the Ethical Committee-Health and Medical Services Group (HMS), Dubai (Reference number-HMS 2059) and written informed consents were obtained from all patients.

### 2.2 Sample size

The sample size was appraised according to the following formula (34): *N* = (Z*_α_*_/2_ s/d)^2^.

Where:

• *N* is the needed sample,
• “*Z_α/2_*” is the normal deviate for a two-tailed alternative hypothesis at a level of significance (Z*_α_*_/2_ equal to 1.96 at an error rate of 0.05%)
• “*s*” is the standard deviation (SD = 15%), and
• “*d*” is the accuracy of estimate or how close it is to the true mean of the main outcome (i.e., margin of error), which is the QPT value after ACLR.

Given the pioneering nature of this study, “*s*” and “*d*” data were collected from a previous work exploring various factors influencing reinjury risk after ACLR, including quadriceps strength outcomes (35). The study provided valuable insights into quadriceps strength outcomes in adult patients undergoing ACLR (35). In this study, the mean QPT was around 85% of the uninjured limb, with a SD of approximately 15%. The margin of error “*d*” was assumed at 4.5%.

The appraised sample size as *N* = (1.96 × 15/4.5)^2^ gives a sample of 42 participants.

### 2.3 Participants

**Figure 1:**
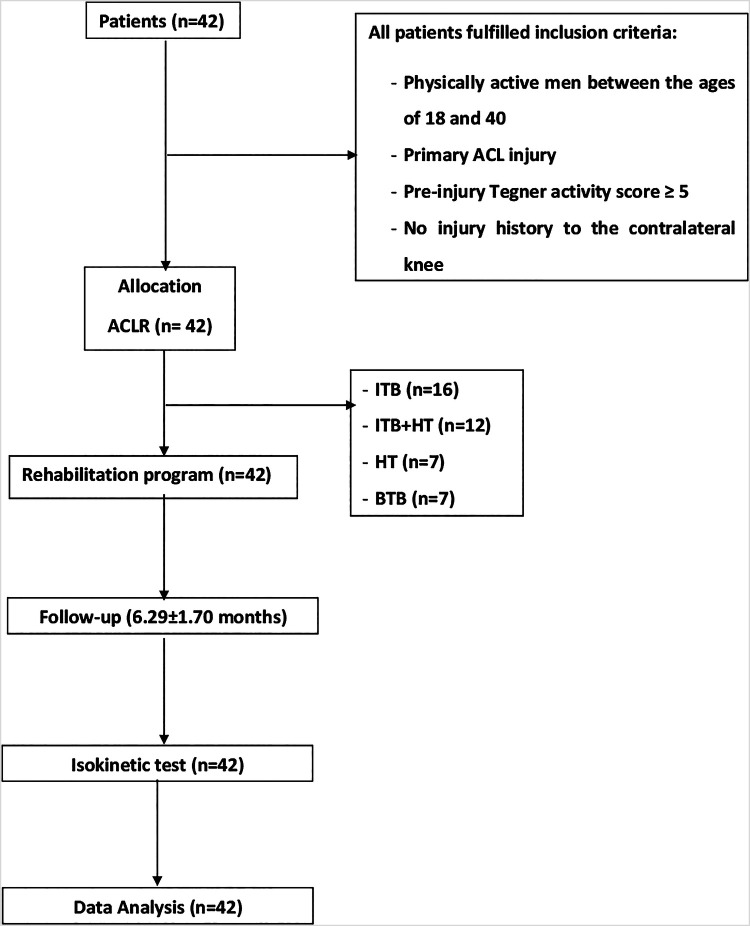
Study flowchart. ACL, anterior cruciate ligament reconstruction; BTB, bone tendon bone; HT, hamstring tendons; ITB, iliotibial band.

The study recruited forty-two active males aged between 18 and 40 years who had sustained a primary ACL injury. Participants were randomly assigned to the four graft groups using a computer-generated randomization sequence created with REDCap (Research Electronic Data Capture), a widely used software for randomization and data management in clinical research. Randomization was stratified by age and activity level to ensure balanced distribution across groups. The allocation sequence was concealed until the intervention assignment to minimize bias (Figure 1):

(i) IITB group (*n* = 16): Primary ACLR employing the ITB.
(ii) ITB + HT group (*n* = 12): Primary ACLR utilizing the ITB with HT augmentation.
(iii) HT group (*n* = 7): Primary ACLR employing HT.
(iv) BTB group (*n* = 7): Primary ACLR utilizing BTB.

### 2.4 Rehabilitation protocol

Participants underwent a comprehensive and standardized ACLR rehabilitation program, consisting of four distinct phases, each tailored to address specific rehabilitation objectives (Box 1). The standardized rehabilitation protocol was applied uniformly across all participants to control for rehabilitation variables and focus on the comparative efficacy of each autograft type.

Box 1 **Rehabilitation protocol.**

**Table:** 

Phase	Duration (weeks)	Description
1	*1*–*2*	Focus on minimizing hemarthrosis and edema. Achievement of full knee range of motion. Restoration of quadriceps muscle control. Normalization of walking.
2	*3*–*6*	Emphasis on passive and active full Range Of Motion (ROM) and full weight-bearing. Introduction of closed kinetic chain exercises with limited (0–50 degrees) ROM. Incorporation of straight-leg raises (extension, flexion, abduction, adduction) and balance/proprioceptive training.
3	*7*–*12*	Progressive increase in closed kinetic chain exercises. Intensification of balance/proprioceptive training. Introduction of frontal and lateral step-ups, lunges with weights, slide board exercises, and stair-master activities.
4	*13*–*26*	Continued progression with closed kinetic chain exercises and initiation of open kinetic chain exercises with full ROM. Intensification of balance/proprioceptive training. Commencement of running and plyometric xercises, cycling, cuttings, and sports-specific drills. Implementation of resistive hip and knee strengthening, plyometric drills, running drills, and balance exercises for both limbs, conducted three days per week.

### 2.5 Strength testing

Isokinetic muscle strength testing was performed in phase 4 using a Biodex isokinetic dynamometer at an angular velocity of 60⁰/s. This velocity was chosen as it provides an optimal balance between assessing muscle strength and maintaining joint safety, minimizing strain on the reconstructed ligament. It is a standard parameter widely used in clinical and research settings for evaluating lower limb strength recovery after ACL reconstruction, as supported by prior studies.

Before testing, participants underwent a standardized warm-up session consisting of ten minutes of low intensity cycling. Each participant performed three maximum-effort quadriceps and hamstring contractions with each leg. QPT and HPT values were recorded, and H:Q ratios were calculated. Stabilization straps were employed to minimize extraneous movement, and the range of motion (ROM) of the knee joint during testing was set from 0 to 100°. Peak torque deficits between the injured and non-injured legs were quantified to evaluate recovery progress.

### 2.6 Statistical analysis

The data set was characterized by SDs and mean values, using the Kolmogorov-Smirnov test to assess normal distribution. A one-way analysis of variance (ANOVA) was used to compare variations between the four groups. A *post hoc* Holm-Bonferroni test was used for pairwise comparison. The effect size was estimated using both Partial Eta squared (*η*²), categorized as trivial (<0.20), small (0.20–0.49), moderate (0.50–0.79), or large (≥0.80) (36) and Cohen's d for pairwise comparisons. Cohen's d was classified as small (0.2), medium (0.5), or large (0.8) (37). The examination of the variability between the healthy knee and the injured knee was carried out by analysis of the graphical method of Bland and Altman. Bland-Altman plots are a powerful graphical tool for comparing two measurement techniques and evaluating the agreement between two sets of data, which the plot provides a visual representation of the difference between two measurements on the *y*-axis and the average of the two measurements on the *x*-axis (38). The Pearson correlation coefficient was used to examine the relationship between healthy and injured leg values.

All statistical analyses were performed using SPSS software version 28.0 (Chicago, IL, USA) and Medcalc software version 20.0 (Ostend, Belgium) for the constrictions of the Bland, Altman graphical method.

## 3 Results

**Figure 2:**
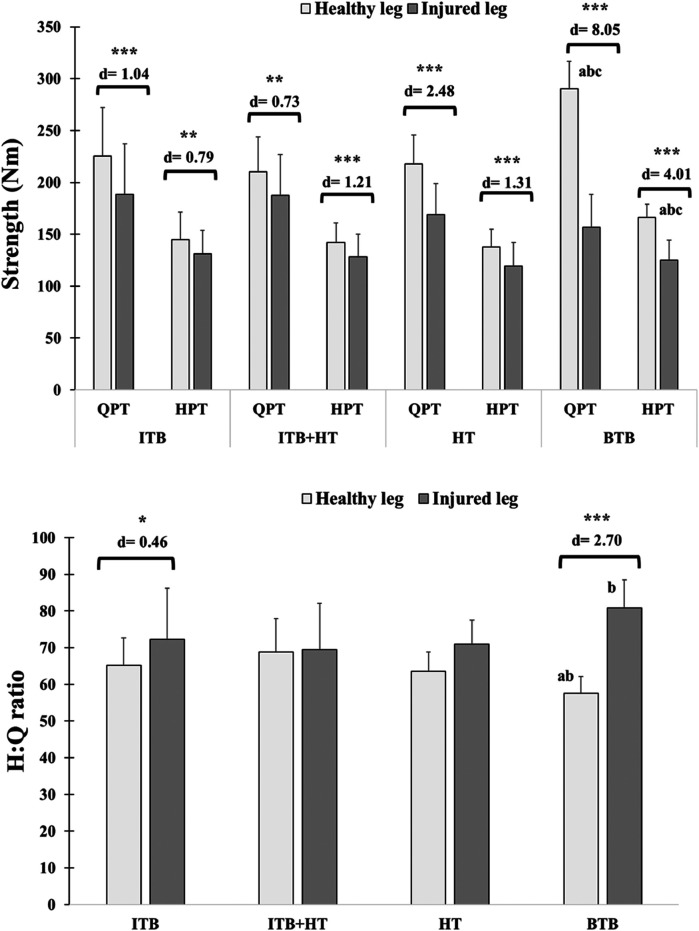
Isokinetic QPT, HPT and H:Q ratio in the healthy and injured legs of ACLR patients across graft types: ITB, ITB + HT, HT, and BTB. Significant differences between the healthy and injured legs are indicated: *P < 0.05, **P < 0.01, ***P < 0.001, with Cohen's *d* values provided as a measure of effect size to indicate the magnitude of these differences. Letters **(a–c)** denote intergroup significant differences. **(a)** BTB vs. ITB; **(b)** BTB vs. ITB + HT; **(c)** BTB vs. HT. BTB, bone patellar tendon bone; HT, hamstring tendons; HPT, hamstring peak torque; ITB, iliotibial band; QPT, quadriceps peak torque.

Significant differences were observed between the BTB group and other groups in height (Cohen's *d* = 1.22–2.01, *P* < 0.05), QPT 's healthy leg (Cohen's *d* = 1.54–2.66, *P* < 0.05), and HPT 's healthy leg (Cohen's *d* = 0.91–1.90, *P* < 0.05). For the H:Q of the healthy leg, differences were significant between the BTB group and both the ITB group (Cohen's *d* = 1.11; *P* < 0.05) and the ITB + TH group (Cohen's *d* = 1.42; *P* < 0.05). Regarding the injured leg, significant differences in the H:Q were identified between the BTB group and the ITB + TH group (Cohen's *d* = 1.03; *P* < 0.05) (Table 1; Figure 2).

**Table 1:** Participants’ characteristics by groups.

Data	Unit/category	ITB (*n* = 16)	ITB + HT (*n* = 12)	HT (*n* = 7)	BTB (*n* = 7)	ANOVA (F)	Effect size
Age	(years)	31.7 (5.6)	32.0 (7.7)	30.3 (6.5)	31.6 (4.3)	0.1	0.01
Height	(cm)	176 (6)	175 (5)	173 (4)	184 (6)^a^^,^^b^^,^^c^	4.9	0.28
Body mass	(kg)	76.2 (11.2)	74.6 (8.3)	75. (6.6)	81.7 (8.3)	0.9	0.07
BMI	(kg/m²)	24,5 (2.4)	24,3 (2.4)	25,3 (2.2)	24,2 (2.2)	0.4	0.03
Following up	(months)	6.3 (1.9)	6.8 (2.1)	5.8 (0.4)	5.6 (0.8)	1.0	0.07

ACL, anterior cruciate ligament; ANOVA, analysis of variance; BMI, body mass index; BTB, bone tendon bone; HT, hamstring tendons; ITB, iliotibial band.

Data were presented as mean (standard deviations).

One-way ANOVA test was used for comparison between the four groups (*P* < 0.05).

^a^ BTB vs. ITB.

^b^ BTB vs. ITB + HT.

^c^ BTB vs. HT.

Regarding comparisons between healthy and injured legs, remarkable differences (*P* < 0.001) within each group were found, and their Cohen's d varied between 0.73 and 8.05. The differences in H:Q ratios between healthy and injured legs are also shown for the ITB group (*P* < 0.01; *d* = 0.46) and BTB (*P* < 0.001; *d* = 2.70) (Figure 2).

**Figure 3:**
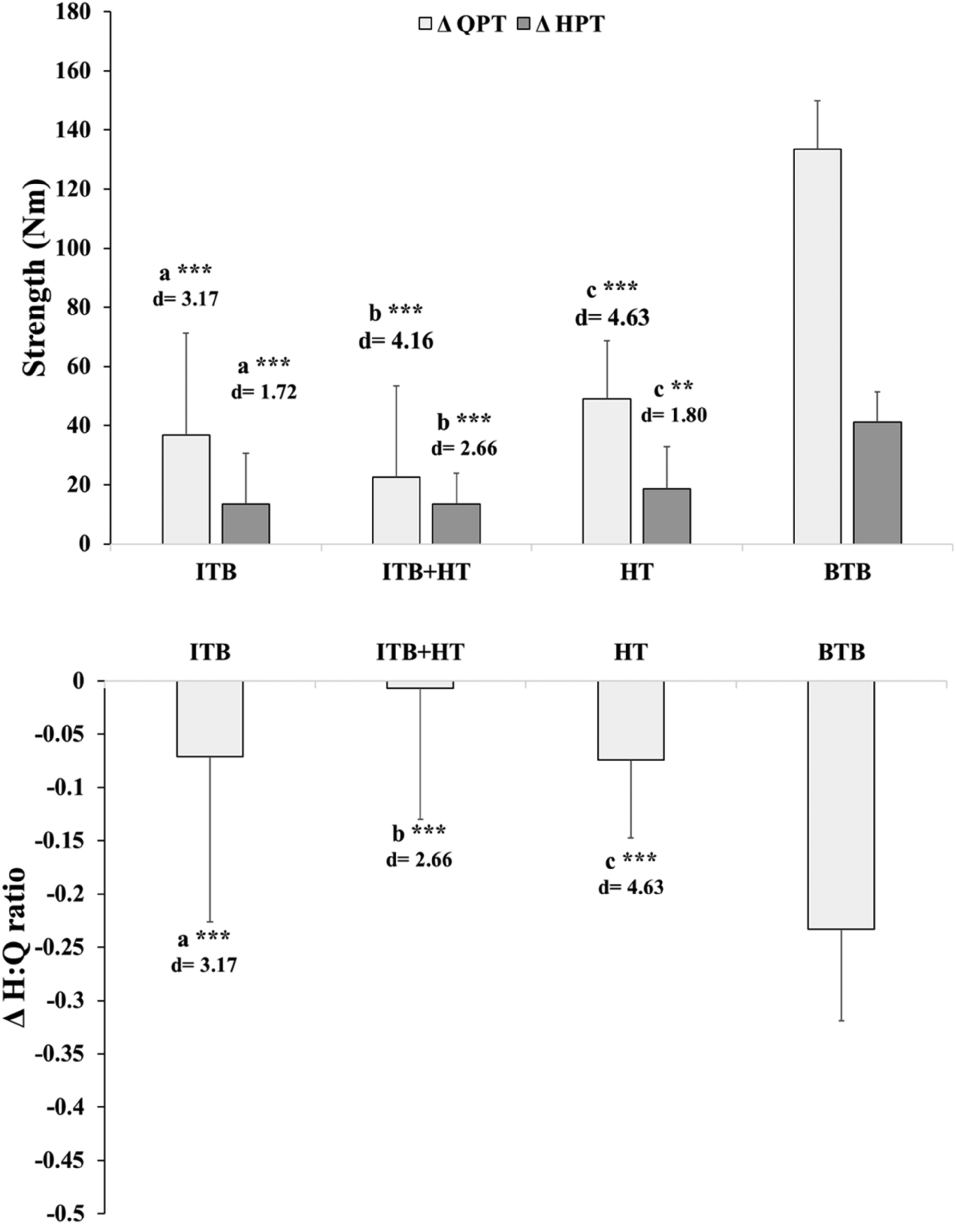
Change (Δ) in quadriceps peak torque (QPT) and hamstring peak torque (HPT) between healthy and injured legs across graft groups: ITB, ITB + HT, HT, and BTB. **P < 0.01, ***P < 0.001, with Cohen's *d* values provided as a measure of effect size to indicate the magnitude of the differences. Letters **(a–c)** denote intergroup significant differences. **(a)** BTB vs. ITB; **(b)** BTB vs. ITB + HT; **(c)**: BTB vs. HT. BTB, bone patellar tendon bone; HT, hamstring tendons; HPT, hamstring peak torque; ITB, iliotibial band; QPT, quadriceps peak torque.

The data analysis focuses on comparing the changes between healthy and injured legs within four groups undergoing ACL surgery. The post-surgical change between the healthy leg and the injured leg appeared significantly higher in the BTB group (*P* < 0.001) compared to the other groups in QPT (Cohen's *d* = 3.17–4.63), HPT (Cohen's *d* = 1. 79–2.66) and H:Q ratios (Cohen's *d* = 1.16–2.03) (Table 2; Figure 3).

**Table 2:** Comparisons of variations (*Δ*) in QPT, HPT and H:Q ratio (healthy leg/injured leg) between the four groups.

Data	Unit/Category	ITB (*n* = 16)	ITB + HT (*n* = 12)	HT (*n* = 7)	BTB (*n* = 7)	F	ANOVA *P*-value	Effect size
Δ QPT	(Nm)	37 (35)	22 (31)	49 (20)	133 (17)	23.77	<0.001	0.65
Δ HPT	(Nm)	13 (17)	13 (10.)	19 (14)	41 (10)	7.27	<0.001	0.37
Δ H:Q ratio	–	−0.07 (0.16)	−0.02 (0.12)	−0.07 (0.07)	−0.23 (0.09)	4.81	0.006	0.28

ANOVA, analysis of variance; BTB, bone patellar tendon bone; HT, hamstring tendons; HPT, hamstring peak torque; ITB, iliotibial band; QPT, quadriceps peak torque.

Data were presented as mean (standard deviations).

One-way ANOVA test was used for comparison between the four groups.

**Figure 4:**
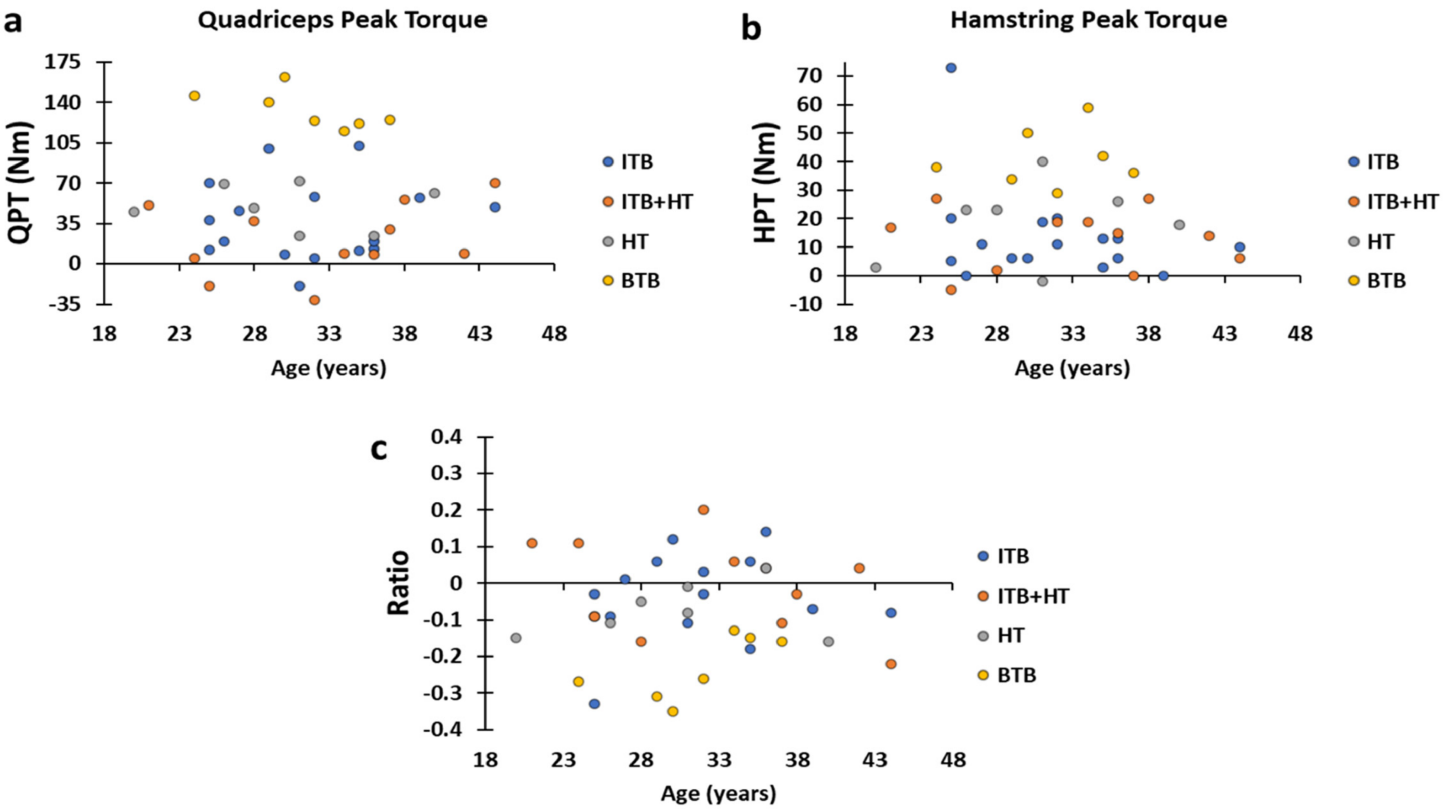
**(a–c)** graphs present the distributions of the variation values of QPT, HPT and the H:Q ratio of the four groups, respectively. BTB, bone patellar tendon bone; HT, hamstring tendons; HPT, hamstring peak torque; ITB, iliotibial band; QPT, quadriceps peak torque.

The distributions of post-surgical change values between the healthy leg and the injured leg identified that the highest are those of group BTB, then we find in second order those of groups ITB and HT, and finally that of ITB + HT, which tends towards zero in QPT and HPT. While the BTB H:Q ratios values vary more than those of the other groups which are scattered on either side of the zero value (Figure 4).

Analysis of the Bland and Man graphs showed:

### 3.1 QPT

**Figure 5:**
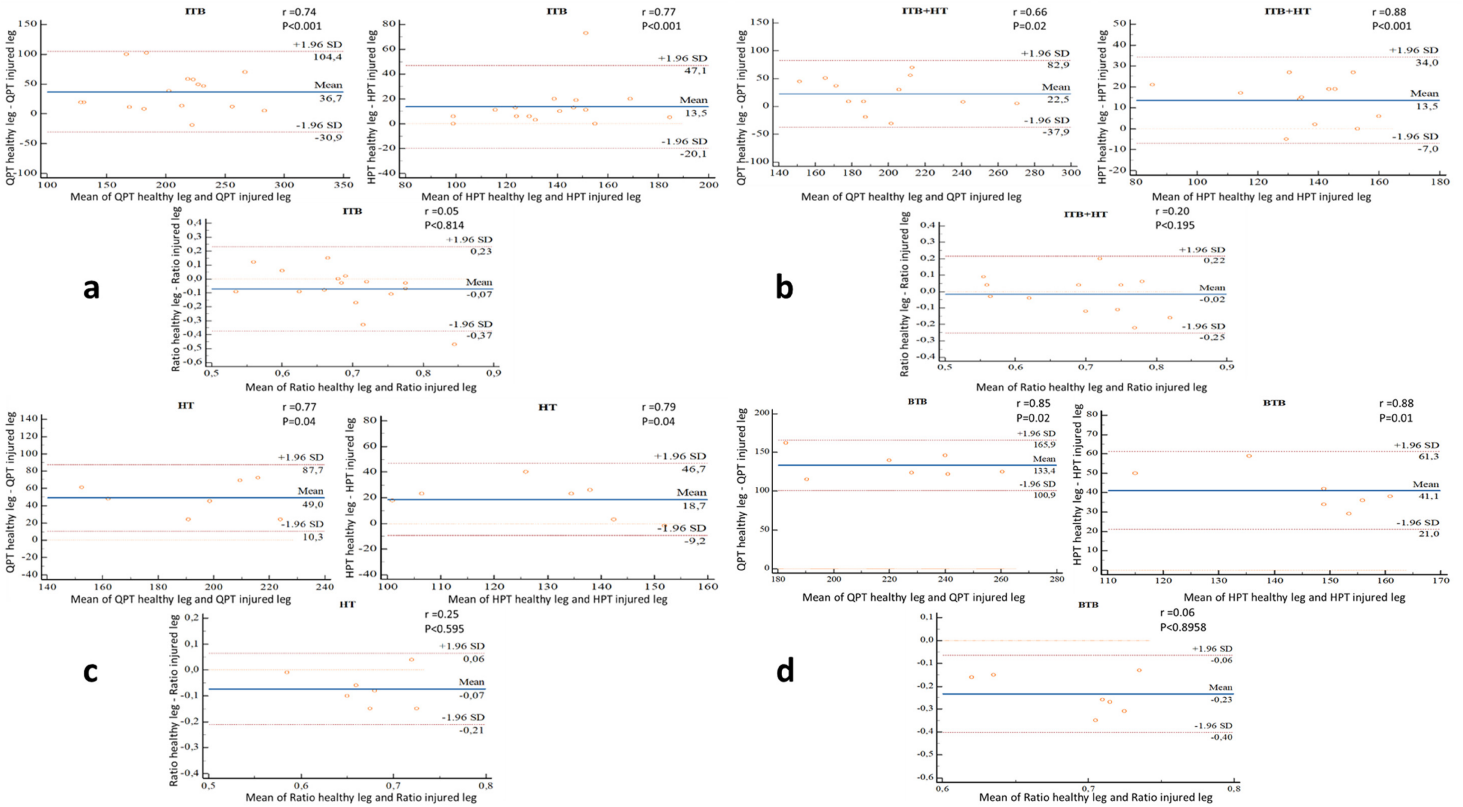
**(a–d)** bland and Man graphs illustrate the different variations (healthy leg/injured leg) of the QPT, HPT and the H:Q ratio of the ITB (*n* = 16), ITB + TH (*n* = 12), TH (*n* = 7) and BTB (*n* = 7) groups respectively with the Pearson correlation coefficients (r). BTB, bone patellar tendon bone; HT, hamstring tendons; HPT, hamstring peak torque; ITB, iliotibial band; QPT, quadriceps peak torque.

The BTB group had the highest mean difference of QPT between the healthy leg and the injured leg of + 133.4 Nm, and an upper-limit of agreement of 165.9 and lower-limit of 100.9 Nm. While for the ITB groups, ITB + HT and HT have mean differences and limits of agreement of 36.7 (upper-limit = 104.4; lower-limit = −30.9), 22.5 (upper-limit = 82.9; lower-limit = −37.9) and 49.6 (upper-limit = 87.7; lower-limit = 10.3), respectively (Figure 5).

### 3.2 HPT

The BTB group had the highest mean difference of HPT between the healthy leg and the injured leg of + 41.1 Nm, and an upper-limit of agreement of 61.3 and lower-limit of 21.0 Nm. While for the ITB groups, ITB + HT and HT have mean differences and limits of agreement of 13.5 (upper-limit = 47.1; lower-limit = −20.1), 13.5 (upper-limit = 34; lower-limit = −7) and 18.7 (upper-limit = 46.7; lower-limit = −9.2), respectively (Figure 5).

### 3.3 H:Q ratio

The BTB group had the highest mean difference of H:Q ratio between the healthy leg and the injured leg of −0.23 and an upper-limit of agreement of −0.6 and lower-limit of −0.40. While for the ITB groups, ITB + HT and HT have mean differences and limits of agreement of −0.7 (upper-limit = 0.23; lower-limit = −0.37), −0.02 (upper-limit = 0.22; lower-limit = −0.25) and −0.07 (upper-limit = 0.6; lower-limit = −0.21), respectively (Figure 5).

The Pearson correlation coefficients between the healthy leg and the injured leg varied from 0.66 (*P* < 0.001) to 0.88 (*P* < 0.001) for QPT and HPT of 4 groups while for H:Q ratio, the coefficients are very low (varied between 0.05 and 0.25) (Figure 5).

## 4 Discussion

Our study investigated the recovery of quadriceps and hamstring strength in ACLR patients with different graft types and revealed nuanced differences. Our findings suggest that while graft selection determines the initial trajectory of muscle strength recovery, the standardized rehabilitation protocol ensured consistent care across all participants.

Specifically, our results identified that patients in the BTB group with an angular velocity of 60⁰/s had more pronounced strength deficits in the quadriceps (45%) and hamstrings (25%) than patients with other grafts such as ITB (16%, 10%), ITB + HT (11%, 10%) and HT (21%, 14%). These results are consistent with a meta-analysis by Shi et al. (39), which reported that BTB patients had greater deficits in extensor mechanism strength but lower deficits in flexor mechanism strength compared to patients with hamstring grafts.

Our research demonstrated significant variations in muscle strength recovery between graft types, attributable to biomechanical and physiological parameters. Quadriceps deficiencies in BTB grafts may arise from the direct effects of graft harvesting and concomitant anterior knee discomfort, hindering optimal muscle activation throughout rehabilitation. In contrast, HT grafts are more prone to impairing hamstring strength due to tissue excision, although they generally maintain quadriceps function more effectively.

These findings emphasize the necessity of customizing rehabilitation methods to address the deficiencies linked to each graft type. BTB graft patients may benefit from early quadriceps-strengthening workouts to alleviate persistent deficiencies, but HT graft patients may necessitate targeted hamstring training to re-establish balance and stability. Customized strategies, underpinned by ongoing assessment of muscular strength recovery, including the H:Q ratio, are essential for enhancing functional results and mitigating re-injury risks (39).

Similarly, Gobbi et al. (40) observed differences in isokinetic performance between BTB and HT grafts in the third and 12th post-operative months at angular velocities of 60°/s, 180°/s, and 300°/s and found a 23% deficit in the quadriceps in BTB patients at the third month after surgery, while HT patients showed deficits primarily in the flexor muscles. However, there were no significant differences between the two groups at the one-year follow-up examination.

In the ongoing controversy about knee muscle strength after ACLR with HT grafts, Manchado et al. (41) pointed out differences in flexor muscle deficits between patients, with the BTB group showing higher values in the operated knee than in the non-operated knee, while the hamstring group showed significant deficits. Morris et al. (42) confirmed these results and found deficits in maximal torque and rate of torque development at various knee joint angles in athletes with ACLR using HT autograft technique compared to the contralateral limb. These findings results are consistent with previous studies indicating significant deficits in BTB reconstructions compared to the contralateral side and support the ongoing discourse on optimal graft selection for ACLR (43).

Divergences between our findings and existing literature can be attributed to several factors, including variations in study populations (age, sex, activity levels, and injury severity) (44–46), differences in rehabilitation protocols, and psychological elements such as readiness to return to sport and kinesiophobia, which affect rehabilitation adherence and performance (45, 47).

The type of graft used in ACLR can have a significant impact on the strength of the quadriceps and hamstring muscles. In our study, all four graft techniques showed deficits in quadriceps strength relative to hamstring strength and the BTB technique had the most notable deficits. In this way, some studies have found that HT grafts can lead to a deficit in quadriceps strength, especially when the knee is flexed while BTB grafts can lead to a deficit in hamstring strength due to altered biomechanics and postoperative adjustments, they can also lead to weakness of the quadriceps due to the involvement of the patellar tendon in the grafting process (48–51). Huber et al. (49) showed that postoperative recovery of thigh muscle function appears to be better with BTB grafts than with HT grafts. According to Tashiro et al. (50), HT leads to significant weakness of hamstring muscle strength at high knee flexion angles. A more recent study has shown that the use of sciatic nerve blockade for ACLR in patients with HT and BTB grafts affects persistent deficits in knee flexor muscle strength at the time of recovery exercise (52). These muscle strength deficits after ACLR are multifactorial. The most important factors include atrophy of the quadriceps due to postoperative immobilization, arthrogenic muscle inhibition due to impaired neuromuscular signaling, and graft-specific effects at the donor site. In addition, joint effusion and swelling impair neuromuscular function, while structural changes, such as cartilage damage, further exacerbate muscle weakness (25, 53). Cartilage damage in particular exacerbates these deficits by altering joint biomechanics and impairing proprioception, which disrupts coordinated muscle activation. The associated pain and joint instability often lead to reduced activity levels, which further accelerates muscle atrophy. These combined factors emphasise the need for targeted rehabilitation protocols that address both the primary effects of surgery and the secondary effects of structural damage. Such measures are crucial for attenuating muscle weakness and restoring optimal joint function after ACLR (54).

These deficits can affect the balance between the quadriceps and hamstrings in the operated legs. The H:Q ratio takes into account the function of two opposing (agonist-antagonist) muscle groups and is the most commonly used parameter to assess muscle strength balance (55–57). Athletes with a H:Q ratio of less than 0.60 have a higher risk of lower limb injury (56, 58). The healthy and injured legs of all groups examined in the present study had an average H:Q ratio above 0.60, which is considered “normal”, except for the healthy leg of the BTB group, which had a value of 0.57. This could be due to pre-existing muscular imbalances or functional deficits that may have been exacerbated by compensatory mechanisms and reduced activity levels following the ACL injury (59). Moreover, anterior knee pain and quadriceps inhibition, often associated with BTB grafts, may affect daily movements and long-term strength balance in the healthy leg (60). In contrast, targeted rehabilitation likely improved the H:Q in the injured leg so that the healthy leg was less considered in recovery protocols (61). This emphasises the importance of bilateral strength training in ACL rehabilitation. In addition, the values of the H:Q ratio of the injured leg are higher than those of the healthy leg in the ACL. This can be observed especially in the first postoperative months, suggesting a possible influence of the graft choice on this H:Q ratio. This imbalance could affect the stability of the knee, as a strong quadriceps without equally strong hamstrings can put more stress on the ACL, increasing the risk of re-injury (62). A higher Q:H ratio in the injured leg indicates that more focus might be needed on strengthening the hamstrings to achieve a more balanced ratio.

We also found in our results that the Q:H ratio of the BTB technique is more deficient than the other techniques. Here are some possible reasons for this observation:

(i) Influence of surgical technique: In BTB grafting, a portion of the patellar tendon is harvested along with bone pegs from the patella and tibia. This procedure can lead to a more pronounced quadriceps weakness, as the patellar tendon is directly involved in the function of the quadriceps. In addition, the altered biomechanics resulting from harvesting the graft may indirectly affect hamstring strength. This highlights the complex interplay between the quadriceps and hamstring and the need for balanced rehabilitation protocols to address deficits in both muscle groups (48–51).;
(ii) Post-operative rehabilitation: BTB graft patients may experience more anterior knee pain and difficulty activating the quadriceps in the early stages of rehabilitation, which may contribute to prolonged quadriceps weakness (63);
(iii) Long-term muscle adaptation: BTB grafts could lead to more significant and longer-lasting quadriceps deficits, while other techniques could allow for a faster and more balanced recovery of muscle strength (64).

The variability in ACL rehabilitation outcomes further emphasizes the importance of considering patient characteristics, surgical techniques, and individualized protocols. These elements, combined with psychological readiness and adherence, play critical roles in optimizing recovery and enabling a return to peak athletic performance (45, 65, 66).

### 4.1 Implications for practice

Since BTB grafts can lead to major deficits in the quadriceps, rehabilitation programs for these patients should include specific protocols aimed at alleviating this imbalance. The focus should be on quadriceps strengthening exercises, ensuring that hamstring strength is also adequately developed. Regular monitoring of the Q:H ratio throughout the rehabilitation process can help to adjust rehabilitation protocols to address any imbalances that may occur. This is critical for all types of grafts but may require more attention in patients with BTB grafts.

### 4.2 Strength and limitation

The present study has several important strengths, including a comprehensive assessment of isokinetic muscle strength and its relationship to graft choice, and a robust design that supports meaningful clinical findings. However, the lack of direct inclusion of patient-reported outcomes, activity levels and a longer follow-up period in the present analysis limits the scope of the conclusions. In addition, the modest sample size, possible variations in surgical techniques and the lack of detailed rehabilitation parameters are limitations. Additionally, while the use of a standardized rehabilitation protocol allows for controlled comparisons of autograft types, it does not account for individual variations in rehabilitation adherence and response. Future studies could explore the interaction between different rehabilitation protocols and graft types to provide a more comprehensive understanding of their combined effects on muscle strength recovery. Nevertheless, the results provide a valuable basis for future research and clinical applications in orthopedic sports medicine.

## 5 Conclusion

This study provides a comprehensive analysis of the impact of different autograft choices (i.e., ITB, ITB + HT, HT, BTB) on the recovery of quadriceps and hamstring muscle strength following anterior ACLR. Our findings highlight significant variations in muscle strength recovery and H:Q ratios among the graft types. The BTB graft demonstrated the most pronounced differences in isokinetic muscle strength between the healthy and injured legs, particularly in the short term. This variability emphasizes the necessity for ongoing evaluation of knee stability and function over a longer period to determine the optimal autograft choice for ACLR. Such long-term assessments are crucial for ensuring sustained muscle strength recovery and overall knee health. The study's results suggest that while all graft types contribute to muscle strength recovery post-ACLR, the HT graft may offer advantages in balancing muscle strength and potentially enhancing knee stability. However, the observed differences in QPT and HPT recovery between graft choices indicate that the selection of the appropriate autograft should be tailored to the individual's specific needs and rehabilitation goals.

## Data Availability

The raw data supporting the conclusions of this article will be made available by the authors, without undue reservation.
